# Marine Peptides from *Solenocera crassicornis* Are Associated with Improved Metabolic, Hepatic, and Intestinal Markers During Diet Normalization in HFD-Induced Obese Mice

**DOI:** 10.3390/nu18122029

**Published:** 2026-06-22

**Authors:** Huirong Lv, Jiaxin Liu, Zhongcang Qian, Gen Lin, Zhengshun Wen

**Affiliations:** 1 Xianghu Laboratory, Hangzhou 311231, China; 2 School of Food Science and Pharmaceutics, Zhejiang Ocean University, Zhoushan 316022, China; 3 Institute of Ecological and Environmental Sciences, Taizhou Academy of Agricultural Sciences, Taizhou 318099, China; 4 Hangzhou Nutrition Biotechnology Co., Ltd., Hangzhou 311200, China

**Keywords:** *Solenocera* *crassicornis*, marine bioactive peptides, antioxidant activity, obesity, gut–liver axis

## Abstract

**Background/Objectives**: Obesity-associated metabolic dysfunction involves oxidative stress, gut barrier impairment, and gut–liver axis disruption. This study evaluated whether enzymatically prepared *Solenocera crassicornis* peptides (SCPs) provide additional benefits during diet normalization in HFD-induced obese mice and examined associations with antioxidant, microbial, and barrier markers. **Methods**: SCPs were characterized using UPLC-Q-TOF-MS/MS and amino acid analysis. Peptides underwent bioactivity prediction and Keap1 docking. After 7 weeks of HFD feeding, obese male C57BL/6J mice were switched to a normal diet and administered vehicle, orlistat, or SCPs for 4 weeks. Adipose tissue mass, serum lipid profiles, liver histology, hepatic antioxidant status, barrier-associated histological and biochemical markers, and gut microbiota composition were assessed. A simulated digestion–fecal fermentation model was used to assess the effects of fermentation products generated in the presence of digested SCPs on H_2_O_2_-induced oxidative injury and MUC2 secretion in LS174T goblet-like cells. **Results**: SCPs reduced epididymal and perirenal fat, improved serum lipids, improved hepatic steatosis-related morphology and enhanced hepatic antioxidant status. SCPs were also associated with improved intestinal morphology, increased mucin-associated staining, decreased serum diamine oxidase levels and reduced hepatic lipopolysaccharide accumulation. 16S rRNA sequencing showed SCP-associated microbial shifts, with correlations linking taxa to metabolic and barrier markers. Fermentation products generated in the presence of digested SCPs improved oxidative-stress and MUC2-related readouts in LS174T cells. **Conclusions**: During diet normalization, SCPs were associated with additional improvements in adiposity, lipid profiles, hepatic antioxidant status, intestinal barrier readouts, and gut microbiota. These findings support further investigation of SCPs as standardized marine protein hydrolysates, but active components, causal mechanisms, long-term efficacy, safety, and human relevance remain to be established.

## 1. Introduction

Obesity is a major public health challenge and a central feature of metabolic syndrome [1,2]. It is closely associated with dyslipidemia, insulin resistance, non-alcoholic fatty liver disease (NAFLD), chronic low-grade inflammation, oxidative stress, and increased risk of cardiovascular disease and some cancers [3,4]. These pathological processes are interconnected. Excessive adiposity alters lipid flux and inflammatory signaling, whereas hepatic steatosis and oxidative stress further aggravate metabolic imbalance. Therefore, a more informative assessment of obesity-related metabolic dysfunction should include multiple readouts, such as adipose tissue mass, serum lipid profiles, liver injury, hepatic oxidative status, and intestinal barrier-associated indices, rather than relying on body weight alone. Because diet is a major modifiable driver of obesity, food-derived bioactive ingredients that support metabolic recovery have attracted increasing attention.

Lifestyle intervention, particularly dietary correction, remains a foundational component of obesity management. However, long-term adherence and weight-loss maintenance remain challenging, and obesity-associated metabolic alterations may persist after withdrawal of an obesogenic diet or after weight reduction [5]. Pharmacological agents such as orlistat can reduce intestinal fat absorption but may cause gastrointestinal adverse effects during prolonged use [6]. These limitations have stimulated interest in natural compounds, including peptides, polysaccharides, polyphenols, alkaloids, and saponins, as complementary nutritional strategies for metabolic regulation [7,8]. Importantly, when such ingredients are evaluated after withdrawal of a high-fat diet (HFD), their effects should be interpreted as additional benefits during dietary correction rather than as independent reversal of obesity under continued HFD exposure.

High-fat diet (HFD)-induced metabolic dysfunction is strongly associated with oxidative stress and disruption of the gut–liver axis [9,10,11]. Excessive reactive oxygen species (ROS) can overwhelm antioxidant defenses, promote lipid peroxidation and inflammation, and contribute to hepatocellular injury [12]. HFD-induced dysbiosis may also impair the mucus layer and epithelial barrier, increasing intestinal permeability and facilitating the transfer of gut-derived lipopolysaccharide (LPS) to the liver through the portal circulation [13]. Because the liver is continuously exposed to gut-derived nutrients, microbial components, and microbial metabolites, barrier-associated impairment may amplify hepatic oxidative stress and steatosis [14]. Thus, dietary interventions that improve antioxidant status and barrier-associated readouts may provide a plausible strategy for supporting metabolic and hepatic recovery, although direct gut–liver axis causality requires dedicated mechanistic validation.

Marine proteins are attractive substrates for generating food-derived bioactive peptides because enzymatic hydrolysis can produce short sequences with improved solubility, digestibility, and potential biological activity [15,16]. Low-molecular-weight peptides may expose hydrophobic, aromatic or proline-rich residues that contribute to radical-scavenging capacity, lipid–interfacial interactions, and protein-binding potential [17]. Shrimp-derived proteins are particularly relevant because shrimp resources and processing by-products provide protein-rich raw materials for peptide preparation. *Solenocera crassicornis* is a commercially important shrimp species in the East China Sea, and enzymatic hydrolysis of its proteins can generate peptides with potential bioactivity. Previous studies reported that low-molecular-weight peptides from *S. crassicornis* protected mice against cyclophosphamide-induced nephrotoxicity and hepatotoxicity, with antioxidant-related mechanisms proposed in those toxin-induced injury models [18,19]. Despite these findings, key gaps remain. Previous studies on *S. crassicornis* peptides have mainly focused on chemical injury models, and it remains unclear whether SCPs provide additional benefits during diet normalization after HFD-induced obesity. Moreover, the associations among SCP intake, antioxidant status, gut microbiota composition, intestinal barrier-associated markers, and fermentation products affecting MUC2-related readouts have not been systematically investigated.

In this study, SCPs were prepared via enzymatic hydrolysis and characterized by means of peptide sequencing and amino acid analysis. Their antioxidant potential and Keap1-binding plausibility were evaluated using in vitro assays and in silico docking. We then examined whether SCP administration was associated with additional improvements in adipose, serum lipid, hepatic, oxidative-stress, intestinal barrier-associated, and gut microbiota-related readouts in HFD-induced obese mice during diet normalization. In addition, simulated gastrointestinal digestion, fecal fermentation, and LS174T goblet-like cell assays were used to explore whether fermentation products generated in the presence of digested SCPs could improve oxidative-stress and MUC2-related readouts in vitro.

## 2. Materials and Methods

### 2.1. Materials

*Solenocera crassicornis* shrimp were purchased from Zhoushan International Aquatic City, Zhejiang, China. Trypsin, alkaline protease, papain, and neutral protease were obtained from Shanghai Ruiyong Biotechnology Co., Ltd. (Shanghai, China), and pepsin was purchased from Shanghai Aladdin Biochemical Technology Co., Ltd. (Shanghai, China). Commercial assay kits for triglyceride (TG), total cholesterol (TC), low-density lipoprotein cholesterol (LDL-C), high-density lipoprotein cholesterol (HDL-C), alanine aminotransferase (ALT), aspartate aminotransferase (AST), total antioxidant capacity (T-AOC), superoxide dismutase (SOD), glutathione peroxidase (GSH-Px), catalase (CAT), and malondialdehyde (MDA) were purchased from Nanjing Jiancheng Bioengineering Institute (Nanjing, Jiangsu, China). Orlistat was purchased from Shanghai Yuanye Bio-Technology Co., Ltd. (Shanghai, China). The bicinchoninic acid (BCA) protein assay kit was also obtained from this supplier. Diamine oxidase (DAO) and lipopolysaccharide (LPS) detection kits were purchased from Wuhan Huamei Biological Engineering Co., Ltd. (Wuhan, Hubei, China). The reactive oxygen species (ROS) assay kit was purchased from Beyotime Biotechnology Co., Ltd. (Shanghai, China). Glutathione reductase (GR), glutathione S-transferase (GST), bovine bile salt, and the periodic acid–Schiff (PAS) staining kit for cells were obtained from Beijing Solarbio Science & Technology Co., Ltd. (Beijing, China). The human mucin 2 (MUC2) ELISA kit was purchased from Elabscience Biotechnology Co., Ltd. (Wuhan, Hubei, China). Dulbecco’s modified Eagle’s medium (DMEM), fetal bovine serum (FBS), trypsin–EDTA solution, penicillin, and streptomycin were purchased from Gibco BRL (Thermo Fisher Scientific, New York, NY, USA). All other chemicals and reagents were of analytical grade unless otherwise stated.

### 2.2. Preparation, Peptide Identification and Amino Acid Analysis of SCPs

#### 2.2.1. Preparation of SCPs

The preparation was slightly modified from previous enzymatic hydrolysis methods [19]. Frozen shrimp were thawed at 4 °C for 12 h. Shells and heads were removed, and the remaining shrimp meat was collected. The meat was homogenized with distilled water at a 1:2 ratio (*w*/*v*). Enzymatic hydrolysis was performed using alkaline protease at 55 °C and pH 8.0, with an enzyme dosage of 7000 U/g for 6 h. After hydrolysis, the enzyme was inactivated in hot water at 98 °C for 15 min, and the hydrolysate was centrifuged at 4000× *g* rpm for 20 min. The supernatant was further centrifuged at 8000× *g* rpm and 4 °C for 10 min, filtered, concentrated, fractionated through a 3 kDa ultrafiltration membrane, and vacuum freeze-dried. The resulting *Solenocera crassicornis* peptides (SCPs) were stored at −20 °C until further use.

#### 2.2.2. Peptide Identification by UPLC-Q-TOF-MS/MS

Peptide sequences in SCPs were identified using an ultra-performance liquid chromatography system coupled with quadrupole time-of-flight tandem mass spectrometry (UPLC-Q-TOF-MS/MS) system (Waters, Milford, MA, USA). The SCP sample was dissolved at 10 mg/mL, filtered through a 0.22 μm membrane, and injected at a volume of 15 μL. The mobile phases consisted of water containing 0.1% formic acid (phase A) and acetonitrile (phase B). Gradient elution was performed at a flow rate of 0.2 mL/min as follows: 0–2 min, 100% A; 2–5 min, 100–50% A; 5–7 min, 50–30% A; 7–8 min, 30–100% A; and 8–10 min, 100% A. The detection wavelength was set at 214 nm, and the column temperature was maintained at 45 °C. Mass spectrometric analysis was performed under the following conditions: ion scanning range, 50–2000 *m*/*z*; capillary voltage, 3000 V; ion source temperature, 100 °C; cone gas flow rate, 50 L/h; and collision energy, 6/20 eV. Peptide sequences were identified by *de novo* sequencing using PEAKS Studio (version 11.0, Bioinformatics Solutions Inc., Waterloo, ON, Canada), and peptides with average local confidence (ALC) scores ≥ 95% were retained.

#### 2.2.3. Free Amino Acid Analysis

The free amino acid composition of SCPs was analyzed using the Hitachi L-8900 amino acid analyzer (Hitachi High-Tech, Tokyo, Japan). Free amino acid composition was determined as an additional compositional index for marine-derived peptide characterization [20]. Briefly, freeze-dried SCPs were dissolved in distilled water, centrifuged at 18,000× *g* rpm for 20 min at 4 °C, and filtered through a 0.22 μm membrane. The filtrate was subjected to amino acid analysis according to the manufacturer’s instructions, and amino acid contents were expressed as mg/g freeze-dried SCPs.

### 2.3. Chemical Antioxidant Activity

#### 2.3.1. Total Antioxidant Capacity (T-AOC) Assay

The total antioxidant capacity (T-AOC) of SCPs was determined using a commercial T-AOC assay kit according to the manufacturer’s protocol. SCPs were dissolved in distilled water at final concentrations of 2, 4, 6, 8, and 10 mg/mL. Briefly, the sample solution was mixed with the reaction reagents supplied in the kit and incubated for 30 min. The absorbance was measured at 520 nm using a Multiskan FC microplate reader (Thermo Fisher Scientific, Waltham, MA, USA). T-AOC was calculated according to the manufacturer’s instructions.

#### 2.3.2. Reducing Power Assay

The reducing power of SCPs was measured using a previously reported method with minor modifications [21]. SCP solutions at five concentrations (2, 4, 6, 8, and 10 mg/mL) were prepared. Each sample solution (1 mL) was mixed with 1 mL of PBS and 1 mL of 1% potassium ferricyanide, followed by incubation at 50 °C for 20 min. Then, 1 mL of 10% trichloroacetic acid was added, and the mixture was centrifuged at 12,000× *g* for 10 min. Subsequently, 1 mL of supernatant was mixed with 1 mL of ultrapure water and 0.2 mL of 0.1% FeCl_3_, followed by incubation at 50 °C for 15 min. Absorbance was measured at 700 nm.

### 2.4. Cell Culture and Experimental Treatments

#### 2.4.1. Cell Culture

Human colorectal adenocarcinoma LS174T cells were obtained from the Shanghai Institute of Cell Biology, Chinese Academy of Sciences (Shanghai, China). LS174T cells were used as a goblet-like in vitro model for MUC2-related secretory readouts [22]; however, this cancer-derived cell line does not fully recapitulate normal intestinal epithelial physiology. Cells were cultured in high-glucose DMEM supplemented with 10% FBS and 1% penicillin-streptomycin solution at 37 °C in a humidified atmosphere containing 5% CO_2_. Cells were passaged every 3 days, and passages 20–26 were used for experiments.

#### 2.4.2. Cytotoxicity and H_2_O_2_-Induced Oxidative Injury Assays

Cell viability was assessed using the MTT assay with minor modifications from previously reported procedures [23]. Cells were seeded into 96-well plates at a density of 1 × 10^4^ cells/well and cultured for 24 h before treatment. For the cytotoxicity assay, cells were treated with SCPs at 200, 400, 600, 800, 1000, 1200, and 1400 μg/mL for 24 h. For the protective assay, cells were divided into control, oxidative damage, and SCP-pretreated groups. The control group received 150 μL of complete culture medium for 36 h. The oxidative damage group was incubated with 150 μL of complete culture medium for 24 h and then treated with 150 μL of H_2_O_2_ solution (1000 μmol/L, prepared in complete culture medium) for 12 h. The SCP-pretreated groups were pretreated with 150 μL of SCPs at 200, 600, or 1000 μg/mL for 24 h and then exposed to 150 μL of H_2_O_2_ solution (1000 μmol/L) for 12 h. After treatment, MTT solution was added, and absorbance was measured at 490 nm using a microplate reader. Cell viability was expressed as a percentage of the control group.

### 2.5. Animal Experiment

Fifty male C57BL/6J mice, 5 weeks old and weighing 16–18 g, were purchased from Beijing Vital River Laboratory Animal Technology Co., Ltd. (Beijing, China). Mice were housed under specific pathogen-free conditions at 23 ± 2 °C and 55 ± 5% relative humidity under a 12 h light/dark cycle, with five mice per cage. Animals had free access to food and water throughout the experiment. All animal procedures were approved by the Institutional Animal Care and Use Committee of Zhejiang Ocean University (Permit No. 2021038) and were conducted in accordance with the Guidelines for the Care and Use of Laboratory Animals.

After a 1-week acclimation period, mice were randomly assigned using a random number sequence to a normal diet group (ND, *n* = 10) or a high-fat diet (HFD) modeling group (*n* = 40). The ND group was fed a normal control diet containing 10% energy from fat (D12450J), whereas the HFD modeling group was fed a high-fat diet containing 60% energy from fat (D12492) for 7 weeks. Both diets were supplied by Wuxi Fanbo Biotechnology Co., Ltd. (Wuxi, China). Body weight was recorded every 2 days during the modeling period. Obesity induction was considered successful when the average body weight of HFD-fed mice exceeded 1.2-fold that of ND mice.

After the 7-week modeling period, HFD-fed mice were randomly divided into four groups with 10 mice per group: the model group (MOD), positive control group (PC), low-dose SCP group (SCPs-L), and high-dose SCP group (SCPs-H). During the following 4-week intervention period, all HFD-induced obese groups were switched from the HFD to the normal diet, whereas the ND group continued receiving the normal diet. The PC group received orlistat at 30 mg/kg body weight, and the SCPs-L and SCPs-H groups received SCPs at 400 and 800 mg/kg body weight, respectively. Orlistat, SCPs, and vehicle were administered once daily by oral gavage at 10 mL/kg body weight. Orlistat and SCPs were administered once daily by oral gavage at 9:00 a.m. The ND and MOD groups received an equal volume of purified water. Body weight and food intake were recorded every 2 days. Food intake was measured at the cage level every 2 days and expressed as g/mouse by dividing cage-level food consumption by the number of mice per cage. Cumulative food intake during the 4-week administration period was then calculated.

At the end of the intervention, mice were fasted overnight and anesthetized with isoflurane. Blood samples were collected from the retro-orbital venous plexus, after which the mice were euthanized by cervical dislocation under anesthesia. The liver, kidney, spleen, perirenal adipose tissue, epididymal adipose tissue, ileum, colon, and cecal contents were rapidly excised. Samples used for biochemical assays and gut microbiota analysis were snap-frozen in liquid nitrogen and stored at −80 °C until analysis, whereas tissues used for histological examination were fixed as described below.

### 2.6. Biochemical Analysis

Blood samples were allowed to stand at room temperature for 2 h and then centrifuged at 4000× *g* rpm for 10 min at 4 °C. The serum supernatant was collected for biochemical assays. Serum ALT, AST, TC, TG, HDL-C, LDL-C, and DAO levels were measured using commercial assay kits according to the manufacturers’ instructions. For tissue biochemical analysis, liver and colon samples were homogenized in ice-cold normal saline at a ratio of 1:9 (*m*/*v*). The homogenates were centrifuged at 4000× *g* rpm for 15 min at 4 °C, and the supernatants were collected. Total protein concentration was determined using a BCA protein assay kit. Hepatic SOD, GSH-Px, and CAT activities and MDA and LPS levels were measured using corresponding commercial kits. Colonic SOD activity and MDA content were measured using the same procedure. All tissue biochemical results were normalized to protein concentration.

### 2.7. Histopathological Analysis

Epididymal adipose tissue was fixed in fat-specific fixative, and liver tissues were fixed in 4% paraformaldehyde for hematoxylin and eosin (H&E) staining. For Oil Red O staining, fresh liver samples were embedded in optimal cutting temperature (OCT) compound, frozen, sectioned, and stained to assess hepatic lipid deposition. Ileum and colon tissues were divided into two portions, including one portion fixed in 4% paraformaldehyde for H&E staining and the other fixed in Carnoy’s fixative (Solarbio, Beijing, China) for AB-PAS staining. Paraffin-embedded tissues were sectioned at 4 μm thickness and stained according to standard protocols. Histological slides were coded before microscopic evaluation, and representative images were assessed by investigators blinded to group allocation. However, formal semi-quantitative histological scoring, adipocyte-size quantification, hepatic triglyceride quantification, Oil Red O-positive area quantification, colon injury scoring, goblet-cell counting, and AB-PAS-positive area quantification were not performed. Therefore, histological findings were interpreted as representative descriptive observations.

### 2.8. Gut Microbiota Analysis

Cecal content samples were collected under sterile conditions and sent to Shanghai Majorbio Bio-pharm Technology Co., Ltd. (Shanghai, China) for gut microbiota analysis. Microbial genomic DNA was extracted using the Solarbio fecal genomic DNA extraction kit (Beijing, China) according to the manufacturer’s instructions. DNA quality and integrity were evaluated via 1% agarose gel electrophoresis. PCR amplification was conducted targeting the V3–V4 hypervariable region of the bacterial 16S rRNA gene using the universal primers 338F: 5′-ACTCCTACGGGAGGCAGCA-3′ and 806R: 5′-GGACTACHVGGGTWTCTAAT-3′. The PCR products were purified using an AxyPrep DNA Gel Extraction Kit (Axygen Biosciences, Union City, CA, USA) and quantified using the QuantiFluor-ST platform (Promega, Madison, WI, USA). Purified amplicons were pooled in equimolar amounts and used to construct paired-end sequencing libraries using the TruSeq™ DNA Sample Preparation Kit (Illumina, San Diego, CA, USA). Sequencing was performed on the Illumina MiSeq platform to generate 250 bp paired-end reads. Raw paired-end reads were merged based on overlap relationships, followed by quality control and filtering using the QIIME (version 1.9.1) [24]. High-quality sequences were clustered into operational taxonomic units (OTUs) at 97% sequence similarity using the UPARSE (version 7.1). To minimize sequencing-depth bias, samples were rarefied to the minimum sequencing depth retained across all samples before alpha- and beta-diversity analyses. Taxonomic assignment was performed using the Ribosomal Database Project (RDP) classifier (version 2.13) against the SILVA SSU rRNA database (release 138.1) [25,26]. Alpha-diversity indices, including Ace, Chao, Shannon, and Simpson indices, were calculated. Beta-diversity was evaluated using principal component analysis (PCA) and principal coordinate analysis (PCoA). Group-level differences in microbial community structure were assessed using PERMANOVA where applicable. Differential taxa were analyzed using the Majorbio cloud platform (Majorbio Bio-pharm Technology Co., Ltd., Shanghai, China), and *p*-values were adjusted for multiple comparisons using false discovery rate (FDR) correction where applicable. All samples were processed and sequenced in the same analytical batch to reduce potential batch effects. Spearman correlation analysis was performed to evaluate associations between gut microbiota and biochemical parameters.

### 2.9. In Vitro Fecal Fermentation and Cell Treatment

#### 2.9.1. Simulated Gastrointestinal Digestion of SCPs

SCPs were subjected to simulated gastrointestinal digestion according to a standardized static in vitro digestion method with minor modifications [27]. For simulated gastric digestion, SCPs were dissolved in distilled water at 30 g/L, and the pH was adjusted to 2.0 using 1 mol/L HCl. Pepsin was then added at an enzyme-to-substrate ratio of 1:50 (*w*/*w*), and the mixture was incubated in a shaking incubator at 37 °C and 100 rpm for 1 h. For simulated intestinal digestion, the pH of the gastric digest was adjusted to 5.0 using 1 mol/L NaOH. Trypsin and bile salt were added at enzyme/substrate or bile salt/substrate ratios of 1:25 and 1:35 (*w*/*w*), respectively. The pH was further adjusted to 7.5 using 1 mol/L NaOH, and the mixture was incubated at 37 °C for 2 h. The digestion reaction was terminated by boiling for 10 min. The digest was lyophilized and stored at −20 °C until use.

#### 2.9.2. In Vitro Fecal Fermentation

Fresh fecal samples from ND and MOD mice were collected under sterile conditions, diluted with PBS (pH 6.8) at a ratio of 1:10 (g/mL), thoroughly homogenized, and centrifuged to remove large particles. The resulting supernatant was mixed with 40% glycerol and stored at −80 °C as fecal microbiota inocula. The basal fermentation medium was prepared as described in Supplementary Table S1. Three fermentation groups were established, namely, the ND microbiota fermentation group (ND), MOD microbiota fermentation group (MOD), and MOD microbiota fermentation group supplemented with gastrointestinally digested SCPs at 10 g/L (MOD-SCPs). Anaerobic fermentation was performed at 37 °C for 24 h. After fermentation, cultures were autoclaved, centrifuged at 4000× *g* rpm for 10 min, and the supernatants were lyophilized and stored at −20 °C for subsequent cell experiments. The lyophilized fermentation products were used as unfractionated mixtures, and their chemical composition was not further profiled in this study.

#### 2.9.3. Treatment of LS174T Cells with Fermented Products

LS174T cells were divided into five groups, namely, the control group (CON), H_2_O_2_-induced oxidative damage group (H_2_O_2_), ND metabolite group (ND), MOD metabolite group (MOD), and MOD-SCPs metabolite group (MOD-SCPs). Cells in the CON group were cultured in complete DMEM. Cells in the H_2_O_2_ group were treated with 400 μmol/L H_2_O_2_ for 12 h and then incubated with complete DMEM for 24 h. Cells in the ND, MOD, and MOD-SCPs groups were first exposed to 400 μmol/L H_2_O_2_ for 12 h and then treated with lyophilized fermented metabolites from the corresponding fermentation groups at 1000 μg/mL for 24 h. Cell viability was determined using the MTT assay. Intracellular ROS levels were assessed using the DCFH-DA assay. Cellular antioxidant status was evaluated by measuring T-AOC, GSH-Px, GR, GST, and MDA using commercial assay kits according to the manufacturers’ instructions. MUC2-related secretory readouts were evaluated via PAS staining and ELISA.

### 2.10. In Silico Bioactivity Prediction and Molecular Docking

The bioactivity probability of identified peptides was predicted using PeptideRanker (https://peptide.ucd.ie/peptideranker/, accessed on 14 April 2026) [28], and amino acid sequences with activity scores ≥ 0.5 were selected as potential bioactive peptides. The AnOxPePred program (https://services.healthtech.dtu.dk/services/AnOxPePred-1.0/, accessed on 16 April 2026) was used to predict peptide free-radical scavenging capacity [29]. ToxinPred (http://crdd.osdd.net/raghava/toxinpred/, accessed on 16 April 2026) was used to predict the toxicity of screened peptides [30]. The BIOPEP-UWM database (https://biochemia.uwm.edu.pl/biopep/start_biopep.php, accessed on 17 June 2026) and Biopeptide Database (http://www.cqudfbp.net/statistics/subPeptideStatistic.jsp?tableName=antioxidative_peptides, accessed on 17 April 2026) were used to search for previously reported bioactive peptides [31]. Representative peptides with high predicted antioxidative activity and no predicted toxicity were selected for molecular docking. Three-dimensional peptide structures were constructed using ChemDraw and Chem3D software (version 21.0, PerkinElmer, Waltham, MA, USA), optimized, and energy-minimized. The crystal structure of the Kelch domain of human Keap1 (PDB ID: 1U6D) was obtained from the RCSB Protein Data Bank. The receptor and ligand structures were hydrogenated and charged using Chem3D and AutoDockTools (version 1.5.7, The Scripps Research Institute, La Jolla, CA, USA) [32]. The active pocket was defined in the Kelch domain, and peptides were docked using AutoDock. Docking conformations were optimized using a genetic algorithm based on minimized docking energy. Molecular docking results were visualized using PyMOL (version 2.6, Schrödinger, New York, NY, USA) and LigPlot (version 2.2, European Bioinformatics Institute, Hinxton, UK), and intermolecular bonds were displayed.

### 2.11. Statistical Analysis

All experiments were performed in at least three independent replicates unless otherwise stated. Animal data are presented as the mean ± standard deviation (SD), with *n* = 10 mice per group unless otherwise indicated. Statistical analyses were performed using SPSS software version 25.0 (IBM Corp., Armonk, NY, USA). Differences among multiple groups were analyzed via one-way analysis of variance (ANOVA), followed by Duncan’s multiple range test. For body weight and food intake measured over time, repeated-measures ANOVA or two-way ANOVA with time and treatment as factors was used where appropriate. Spearman correlation analysis was used to assess associations between gut microbiota and biochemical parameters. A *p* < 0.05 was considered statistically significant, and *p* < 0.01 was considered highly significant. Graphs were generated using OriginPro 2021 (OriginLab Corporation, Northampton, MA, USA).

## 3. Results

### 3.1. Characterization and Antioxidant Activity of SCPs

The peptide and amino acid composition of SCPs were analyzed via UPLC-Q-TOF-MS/MS. Peptide sequences were identified *de novo* from the mass spectrometry data using PEAKS software, and the results are presented in Table S2. A total of 17 peptides with average local confidence (ALC) scores ≥ 95% were identified. These peptides contained 4–8 amino acids and had molecular weights below 1 kDa. Among them, 15 contained proline, 9 contained alanine, and 9 contained leucine, indicating a high proportion of residues associated with hydrophobicity and peptide conformation. As shown in Table S3, 17 free amino acids were detected in SCPs, including 7 essential amino acids and 6 flavor-associated amino acids. The four most abundant amino acids were arginine (Arg, 34.627 mg/g), phenylalanine (Phe, 25.455 mg/g), glycine (Gly, 24.451 mg/g), and tyrosine (Tyr, 23.467 mg/g). Essential amino acids (Thr, Val, Met, Ile, Leu, Phe, and Lys) accounted for 39.36% of the total amino acid mass, while non-essential amino acids (Asp, Ser, Glu, Gly, Ala, Cys, Tyr, Arg, Pro, and His) accounted for 60.64%.

**Figure 1:**
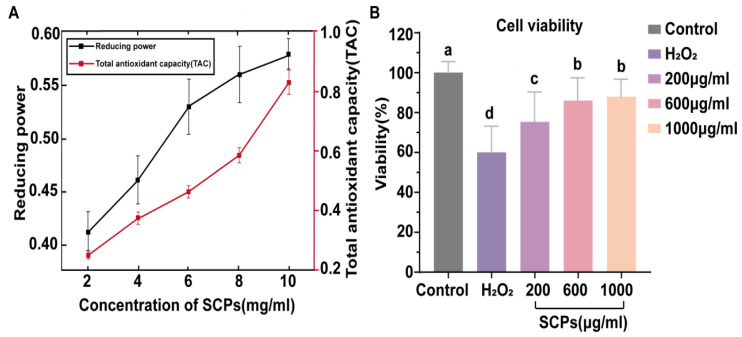
Chemical antioxidant activity of SCPs and their effects on H_2_O_2_-induced oxidative injury in LS174T cells. (**A**) Total antioxidant capacity and reducing power of SCPs at different concentrations. (**B**) Effects of SCP pretreatment on the viability of H_2_O_2_-challenged LS174T cells. Data are expressed as mean ± SD. Different letters indicate significant differences (*p* < 0.05).

In the reducing power assay, higher absorbance at 700 nm indicates stronger electron donation capacity through Fe^3+^ reduction to Fe^2+^. As shown in Figure 1A, SCPs showed dose-dependent reducing power and total antioxidant capacity. At 10 mg/mL, the reducing power reached 0.59, and the total antioxidant capacity reached 0.86. The effect of SCPs on H_2_O_2_-induced oxidative injury in LS174T cells is shown in Figure 1B. Compared with the control group, H_2_O_2_ exposure significantly decreased LS174T cell viability (*p* < 0.05). Pretreatment with low, medium, and high doses of SCPs significantly increased cell viability compared with the H_2_O_2_ group (*p* < 0.05), although there was no significant difference between the 600 and 1000 μg/mL SCP pretreatments (*p* > 0.05). These findings indicate that SCPs exhibited chemical antioxidant activity and improved LS174T cell viability under H_2_O_2_-induced oxidative stress in vitro.

### 3.2. Bioactivity Prediction and Molecular Docking of SCPs

**Figure 2:**
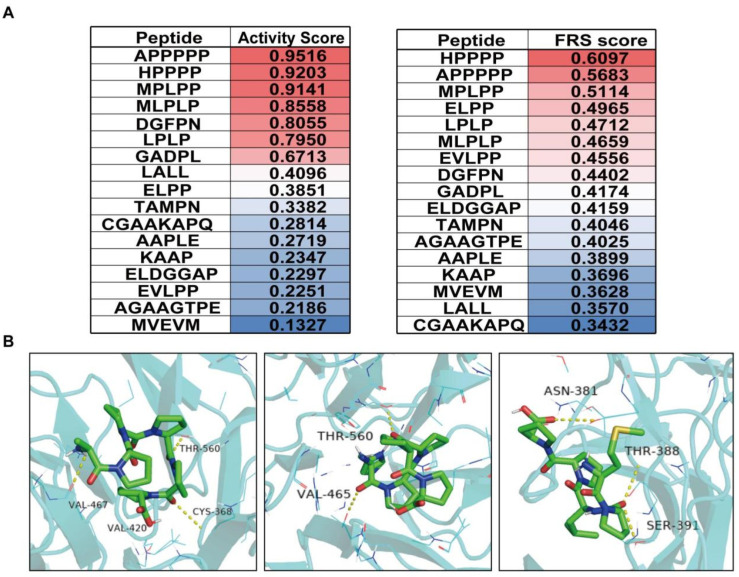
Bioactivity prediction and Keap1 docking of representative peptide sequences. (**A**) Prediction of antioxidant activity of peptides. Peptides are ranked according to activity score (left panel) and free radical scavenging (FRS) score (right panel). The color gradient from red to blue indicates high-to-low predicted values, with red representing higher activity scores and blue representing lower scores. (**B**) Predicted docking interactions of APPPPP, HPPPP, and MPLPP with the Keap1 Kelch domain.

As shown in Figure 2A, the bioactivity probability of the 17 identified peptides (ALC ≥ 95%) was first screened using PeptideRanker. Seven peptides had activity scores ≥ 0.5, suggesting potential bioactivity. The free-radical scavenging probability of the identified peptides was further assessed using AnOxPePred, and three peptides with relatively high predicted scores, HPPPP, APPPPP, and MPLPP, were selected for subsequent in silico interaction analysis. BIOPEP-UWM screening showed that LPLP matched a previously reported antihypertensive peptide with ACE-inhibitory activity, whereas the remaining peptides did not match known bioactive entries under the selected search settings. ToxinPred screening indicated no obvious toxicity risk for HPPPP, APPPPP, and MPLPP. Molecular docking was then used to evaluate the binding plausibility of HPPPP, APPPPP, and MPLPP with the Keap1 Kelch domain. As shown in Figure 2B, APPPPP, HPPPP, and MPLPP formed multiple hydrogen bonds with residues in the Keap1 Kelch domain, including VAL467, VAL420, CYS368, THR560, VAL465, ASN381, THR388, and SER391. These interactions suggest the possible Keap1-binding plausibility of the selected peptides and provide a structural hypothesis for antioxidant-related activity. However, docking evidence alone does not demonstrate Keap1 target engagement, Keap1/Nrf2 dissociation, Nrf2 nuclear translocation, ARE activation, or downstream antioxidant pathway activation, all of which require direct functional validation.

### 3.3. SCPs Improve Fat-Pad Mass and Serum Lipid Profiles

After 7 weeks of HFD feeding, the average body weight of mice in the HFD group reached approximately 32 g, which was 1.23-fold that of mice in the ND group, confirming the successful establishment of the obesity model. During the subsequent 4-week intervention, all HFD-induced obese groups (MOD, PC, SCPs-L, and SCPs-H) were switched to the normal diet. Final body weights after 4 weeks of intervention are shown in Table S4. At the end of the experiment, the average body weight of the MOD group remained significantly higher than that of the ND group (*p* < 0.05). Although body weight decreased after diet normalization, the PC and SCP groups did not differ significantly from the MOD group in final body weight (*p* > 0.05).

During the 4-week administration period, cumulative food intake was summarized to evaluate whether changes in energy intake might contribute to the observed metabolic effects (Figure S1). The MOD group showed lower cumulative food intake than the ND group, whereas the PC group tended to show higher intake than the MOD group. Cumulative food intake in the SCPs-L and SCPs-H groups did not differ significantly from that in the MOD group (*p* > 0.05). These data suggest that the improvements in fat-pad mass and serum lipid markers observed in SCP-treated mice were unlikely to be explained by reduced food consumption alone. However, energy expenditure was not assessed.

Organ weights are shown in Table S5. Liver, kidney, and spleen weights did not differ significantly between the MOD and ND groups (*p* > 0.05). However, epididymal and perirenal adipose tissue weights were significantly higher in the MOD group than in the ND group (*p* < 0.05), and treatment with SCPs or PC significantly reduced these adipose tissue weights compared with the MOD group (*p* < 0.05). These results suggest that SCPs were associated with reduced fat-pad mass during diet normalization, although this was not accompanied by a significant reduction in final body weight relative to the MOD group.

**Figure 3:**
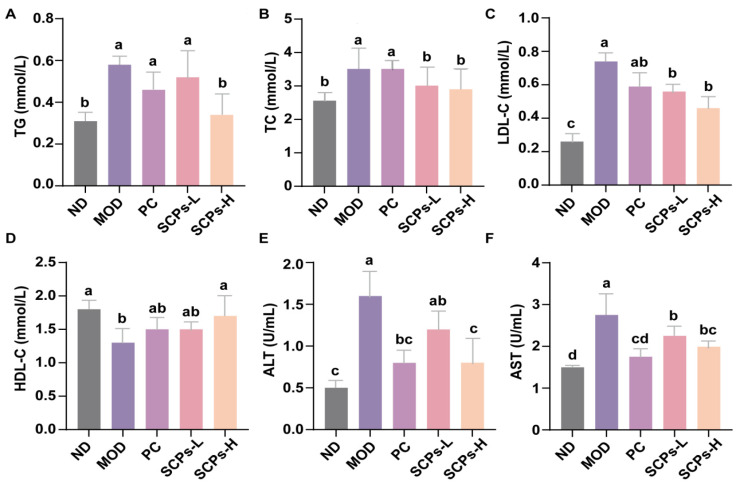
Effects of SCPs on serum lipid profiles and liver function-related biochemical markers in HFD-induced obese mice. Serum levels of (**A**) TG, (**B**) TC, (**C**) LDL-C, and (**D**) HDL-C and activities of (**E**) ALT and (**F**) AST. Data are expressed as mean ± SD (*n* = 10). Different letters indicate significant differences (*p* < 0.05).

As shown in Figure 3, serum TC, TG, LDL-C, ALT, and AST levels were significantly increased in the MOD group compared with the ND group (*p* < 0.05), whereas HDL-C was significantly decreased (*p* < 0.05). Compared with the MOD group, SCPs-H significantly reduced serum TC, TG, LDL-C, ALT, and AST levels and significantly increased HDL-C (*p* < 0.05). SCPs-L did not significantly affect ALT, TG, or HDL-C (*p* > 0.05). Collectively, these results indicate that high-dose SCP administration was associated with improved serum lipid profiles and liver function-related biochemical markers in HFD-induced obese mice under diet normalization.

### 3.4. SCPs Are Associated with Improved Hepatic Steatosis-Related Morphology and Hepatic Antioxidant Status

**Figure 4:**
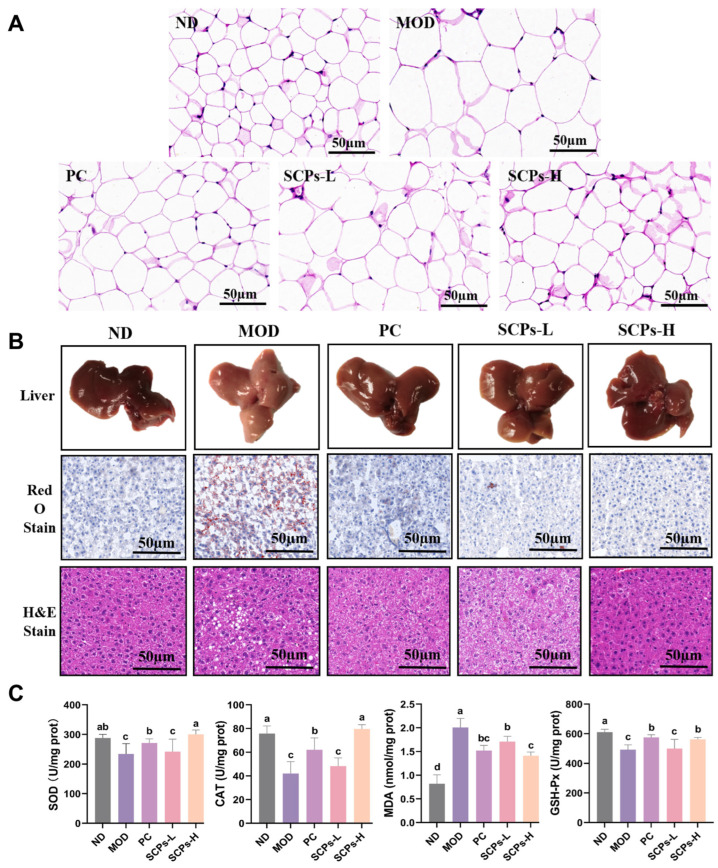
Effects of SCPs on adipose tissue morphology, hepatic lipid accumulation, and hepatic antioxidant status in HFD-induced obese mice. (**A**) Representative H&E staining of epididymal adipose tissue. Scale bar = 50 μm. (**B**) Representative images of liver macroscopic appearance, Oil Red O staining and H&E staining showing hepatic morphology and lipid deposition across experimental groups. Scale bar = 50 μm. (**C**) Hepatic antioxidant markers, including MDA level and the activities of SOD, GSH-Px, and CAT in liver tissues. Data are expressed as mean ± SD (*n* = 10). Different letters indicate significant differences (*p* < 0.05).

H&E staining of epididymal adipose tissue is shown in Figure 4A. Compared with the ND group, the MOD group showed enlarged adipocytes. SCP and PC treatments appeared to reduce adipocyte enlargement compared with the MOD group. These results indicate that SCP intervention was associated with improved adipose tissue morphology during the diet-normalization period.

Macroscopic liver morphology, Oil Red O staining, and H&E staining are shown in Figure 4B. Livers from the ND group appeared dark red, with sharp edges and no visible lipid granules. In contrast, livers from the MOD group were pale red, greasy on the cut surface, and soft in texture, with an indistinct outline. Livers from SCP-treated mice resembled those from the ND group, showing a darker red color, sharper edges, and reduced surface granularity. Oil Red O staining showed marked lipid droplet accumulation in the MOD group compared with the ND group, whereas lipid droplet deposition appeared lower in SCP-treated groups, especially the SCPs-H group, and in the PC group. H&E staining further showed regular hepatocyte morphology and compact arrangement in the ND group, whereas the MOD group exhibited disordered hepatic architecture, diffuse lipid vacuoles of different sizes, and loose cytoplasm. In the SCPs-H group, lipid vacuoles appeared reduced, and hepatic structure was closer to that of the ND group. Representative histological observations suggest that SCPs were associated with improved hepatic steatosis-related morphology in HFD-induced obese mice during diet normalization. These histological findings should be interpreted as representative morphological observations rather than quantitative evidence of reduced adipocyte hypertrophy or hepatic lipid burden.

Hepatic oxidative stress markers are shown in Figure 4C. Compared with the ND group, hepatic MDA content was significantly increased in the MOD group (*p* < 0.05), while GSH-Px, CAT, and SOD activities were significantly decreased (*p* < 0.05). Compared with the MOD group, SCPs-H and PC treatment significantly increased GSH-Px, CAT, and SOD activities and decreased MDA content (*p* < 0.05). These results indicate that SCPs were associated with improved hepatic antioxidant status in HFD-induced obese mice during diet normalization.

### 3.5. SCPs Improve Barrier-Associated Histological and Biochemical Markers

**Figure 5:**
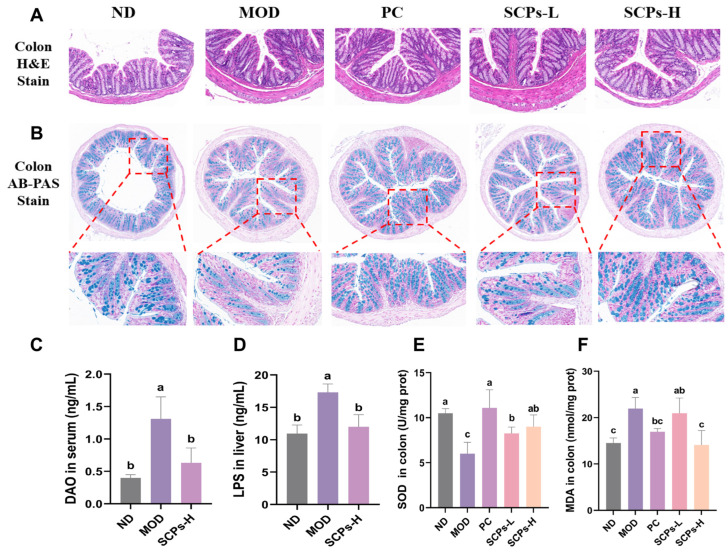
SCPs improve barrier-associated histological and biochemical readouts and mitigate oxidative stress in HFD-induced obese mice. (**A**) Representative H&E staining of colon sections. (**B**) Representative AB-PAS staining of colon sections. (**C**) Serum diamine oxidase (DAO) levels. (**D**) Hepatic lipopolysaccharide (LPS) content. (**E**) Colonic superoxide dismutase (SOD) activity. (**F**) Colonic malondialdehyde (MDA) levels. Data are expressed as mean ± SD (*n* = 10). Different letters indicate significant differences (*p* < 0.05).

Colon histology was evaluated using H&E staining and AB-PAS staining. H&E staining showed normal colonic morphology in the ND group, with relatively intact crypt architecture and no obvious inflammatory infiltration (Figure 5A). In contrast, the MOD group displayed marked mucosal injury, including distorted crypt architecture, epithelial disruption, and inflammatory cell infiltration. PC and SCP treatment alleviated these morphological alterations, with improved crypt structure and reduced inflammatory infiltration compared with the MOD group.

AB-PAS staining was used to evaluate goblet cells and mucin-associated staining in the colon (Figure 5B). The ND group showed abundant goblet cells and strong mucin-associated staining in colonic crypts, whereas the MOD group showed disrupted crypt structure, reduced goblet-cell staining, and weakened mucin-associated staining. PC and SCP treatment were associated with improved crypt morphology and increased goblet-cell/mucin-associated staining compared with the MOD group, with the SCPs-H group showing a more evident improvement than the SCPs-L group.

Serum DAO and hepatic LPS levels were measured as barrier-associated biochemical indices (Figure 5C,D). Compared with the ND group, the MOD group showed significantly increased serum DAO and hepatic LPS levels (*p* < 0.05). SCPs-H treatment significantly reduced both DAO and hepatic LPS levels compared with the MOD group (*p* < 0.05), suggesting improvement in barrier-associated biochemical readouts and reduced hepatic accumulation of gut-derived endotoxin. Colonic oxidative status was assessed by measuring SOD activity and MDA content (Figure 5E,F). Compared with the ND group, the MOD group showed significantly decreased colonic SOD activity and increased MDA content (*p* < 0.05). SCPs-H significantly increased SOD activity and decreased MDA content compared with the MOD group (*p* < 0.05). Collectively, these results indicate that SCPs-H was associated with improved colon barrier-associated histological and biochemical markers and reduced colonic oxidative stress during diet normalization. These colon histological findings should be interpreted as representative descriptive observations supporting the biochemical barrier-associated readouts.

### 3.6. SCPs Are Associated with Shifts in Gut Microbiota Composition

**Figure 6:**
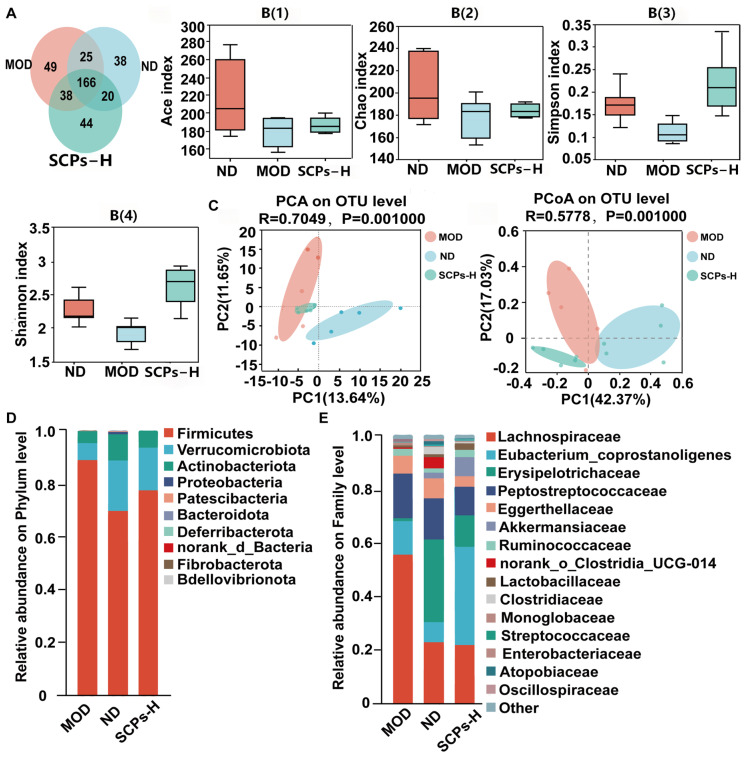

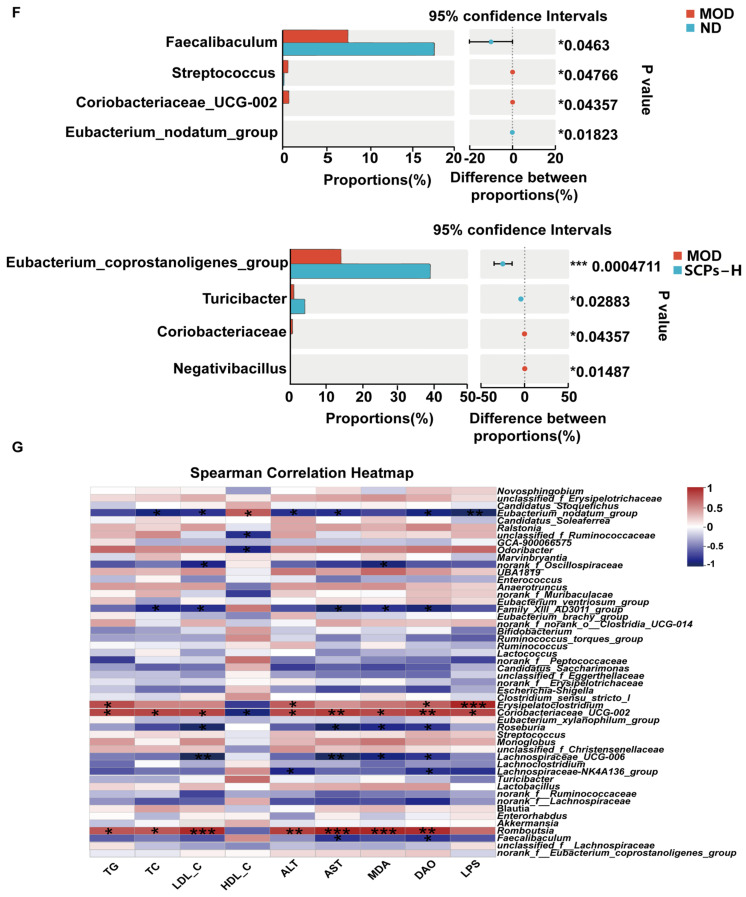
SCPs modulate the composition and structure of the gut microbiota in HFD-induced obese mice. (**A**) Venn diagram showing the overlap of operational taxonomic units (OTUs). (**B**) Alpha diversity indices (Ace, Chao, Shannon, and Simpson). (**C**) PCA/PCoA plots. Relative abundance of gut microbiota at the (**D**) phylum and (**E**) family levels. (**F**) Relative abundance of key differential taxa at the genus level. (**G**) Spearman correlation heatmap between key microbial genera and metabolic parameters. Statistical significance is indicated as * *p* < 0.05, ** *p* < 0.01, and *** *p* < 0.001.

The Venn diagram identified 166 core OTUs shared by all three groups, with 49, 38, and 44 group-specific OTUs in the MOD, ND, and SCPs-H groups, respectively (Figure 6A). Alpha diversity indices are shown in Figure 6B. The MOD group showed lower Chao, Shannon, and Simpson indices compared with the ND group, suggesting that HFD altered microbial richness and diversity. In contrast, SCPs-H treatment partially restored these alpha-diversity indices relative to the MOD group. Beta-diversity was evaluated using principal component analysis (PCA) and principal coordinate analysis (PCoA) at the OTU level (Figure 6C). The MOD group clustered separately from the ND group, revealing an HFD-induced shift in microbial structure. The SCPs-H group diverged from the MOD group, suggesting that SCPs-H administration altered microbial community composition during diet normalization. At the phylum level (Figure 6D), the murine gut microbiota was mainly composed of Firmicutes, Verrucomicrobiota, and Actinobacteria. Compared with the ND group, the MOD group showed an increased relative abundance of Firmicutes and lower relative abundances of Verrucomicrobiota and Actinobacteriota. SCPs-H treatment partially reversed these HFD-induced changes. At the family level (Figure 6E), dominant taxa included Lachnospiraceae, the Eubacterium coprostanoligenes group, Erysipelotrichaceae, and Peptostreptococcaceae. HFD induction enriched Lachnospiraceae and decreased Erysipelotrichaceae, whereas SCP intervention partially corrected these changes. Compared with the MOD group, the SCPs-H group also showed increased relative abundances of the Eubacterium coprostanoligenes group, Akkermansiaceae, and Lactobacillaceae. At the genus level (Figure 6F), the relative abundances of *Faecalibaculum* and the *Eubacterium nodatum* group were significantly lower in the MOD group than in the ND group (*p* < 0.05), whereas *Streptococcus* and *Coriobacteriaceae* UCG-002 were significantly higher (*p* < 0.05). Compared with the MOD group, SCP intervention significantly increased the relative abundances of the *Eubacterium coprostanoligenes* group and *Turicibacter* (*p* < 0.05), while significantly decreasing *Coriobacteriaceae* UCG-002 and *Negativibacillus* (*p* < 0.05).

Spearman correlation analysis was performed to evaluate the associations between specific gut microbiota genera and metabolic parameters, liver function, and intestinal injury markers (Figure 6G). Serum levels of TG, TC, and LDL-C were negatively correlated with the *Eubacterium nodatum* group, *Roseburia*, the Family XIII AD3011 group, and *Lachnospiraceae* UCG-006, but positively correlated with *Romboutsia* and *Coriobacteriaceae* UCG-002. Serum HDL-C levels exhibited a positive correlation with the *Eubacterium nodatum* group and a negative correlation with unclassified *Ruminococcaceae*, *Odoribacter*, and *Coriobacteriaceae* UCG-002. Regarding liver function, *Romboutsia* and *Coriobacteriaceae* UCG-002 were positively correlated with serum AST and ALT levels, whereas the *Eubacterium nodatum* group, the Family XIII AD3011 group, *Roseburia*, *Lachnospiraceae* UCG-006, the *Lachnospiraceae* NK4A136 group, and *Faecalibaculum* showed negative correlations with these markers. Furthermore, indicators of intestinal oxidative stress and permeability (MDA, DAO, and LPS) were positively correlated with *Erysipelatoclostridium*, *Coriobacteriaceae* UCG-002, and *Romboutsia*. In contrast, these markers were negatively correlated with the *Eubacterium nodatum* group, unclassified *Oscillospiraceae*, the Family XIII AD3011 group, *Lachnospiraceae* UCG-006, *Roseburia*, the *Lachnospiraceae* NK4A136 group, unclassified *Ruminococcaceae*, unclassified *Lachnospiraceae*, and *Faecalibaculum*. These findings indicate that SCPs-H administration was associated with partial shifts in gut microbiota composition and correlations with metabolic and intestinal barrier-related markers during diet normalization, but causality cannot be inferred from these associations.

### 3.7. Fermentation Products of Digested SCPs Attenuate Oxidative Stress and Upregulate MUC2 in Cells

**Figure 7:**
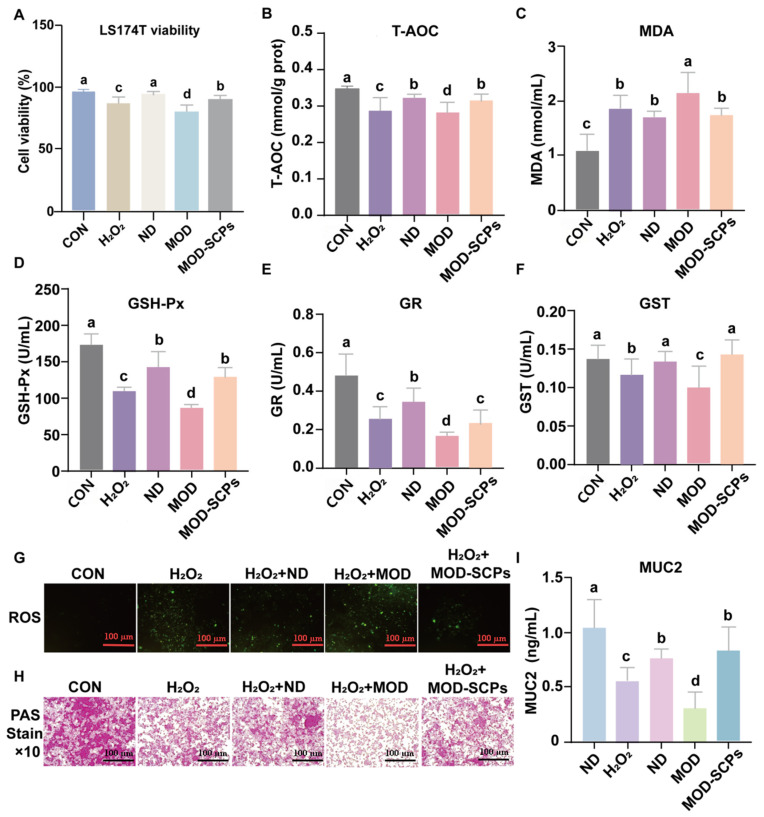
Effects of fermentation products generated in the presence of digested SCPs on H_2_O_2_-induced oxidative injury and MUC2-related readouts in LS174T goblet-like cells. (**A**) Cell viability. (**B**) Total antioxidant capacity (T-AOC). (**C**) Malondialdehyde (MDA) levels. (**D**) Glutathione peroxidase (GSH-Px) activity. (**E**) Glutathione reductase (GR) activity. (**F**) Glutathione S-transferase (GST) activity. (**G**) Intracellular ROS accumulation detected via DCFH-DA fluorescence. Scale bar = 50 μm. (**H**) PAS staining showing mucin-associated glycoprotein staining. (**I**) MUC2 secretion quantified via ELISA. Scale bar = 50 μm. Data are expressed as mean ± SD. Different letters indicate significant differences (*p* < 0.05).

The effects of fermentation products on LS174T goblet-like cells were evaluated after H_2_O_2_-induced oxidative injury. Cell viability, intracellular ROS levels, antioxidant enzyme activities, and MUC2-related readouts were assessed (Figure 7). As shown in Figure 7A, H_2_O_2_ exposure significantly reduced LS174T cell viability compared with the control group (*p* < 0.05). ND fermentation products also improved cell viability, whereas MOD fermentation products further reduced viability. MOD-SCPs’ fermentation products partially restored viability relative to both H_2_O_2_ and MOD fermentation products. Cellular antioxidant status was then evaluated by measuring T-AOC, MDA, GSH-Px, GR, and GST (Figure 7B–F). Compared with the control group, H_2_O_2_ exposure significantly decreased T-AOC and the activities of GSH-Px, GR, and GST, while increasing MDA accumulation (*p* < 0.05). MOD fermentation products further aggravated these oxidative-stress-related changes, as reflected by lower antioxidant enzyme activities and higher MDA levels. Compared with the MOD group, MOD-SCPs’ fermentation products significantly increased T-AOC, GSH-Px, GR and GST activities and reduced MDA content (*p* < 0.05). These results indicate that fermentation products generated in the presence of digested SCPs improved selected antioxidant readouts in H_2_O_2_-challenged LS174T cells.

Consistent with the biochemical data, DCFH-DA fluorescence imaging showed weak ROS fluorescence in control cells and marked ROS accumulation after H_2_O_2_ exposure (Figure 7G). Cells treated with MOD fermentation products exhibited stronger fluorescence than those treated with ND fermentation products, whereas MOD-SCPs’ fermentation products visibly reduced ROS fluorescence compared with the MOD group.

PAS staining and ELISA were used to evaluate MUC2-related secretory readouts (Figure 7H,I). H_2_O_2_ exposure weakened PAS staining and significantly decreased MUC2 secretion compared with the control group (*p* < 0.05). MOD fermentation products further reduced PAS staining intensity and MUC2 levels, whereas MOD-SCPs’ fermentation products increased PAS-positive staining and significantly elevated MUC2 secretion compared with the MOD group (*p* < 0.05). Collectively, these findings suggest that fermentation products generated in the presence of digested SCPs improved oxidative-stress and MUC2-related readouts in this LS174T goblet-like cell model.

## 4. Discussion

The physiological functions of bioactive peptides are closely related to molecular weight, amino acid composition, hydrophobicity, and sequence features. In this study, SCPs prepared via alkaline protease hydrolysis were fractionated below 3 kDa and contained short peptides rich in proline, alanine, and leucine. Such residues may promote hydrophobic interactions at lipid interfaces and contribute to radical scavenging and inhibition of lipid peroxidation [33]. Molecular docking provided structural plausibility that representative sequences (HPPPP, APPPPP, and MPLPP) may interact with the Keap1 Kelch domain through hydrogen bonding. These docking results are consistent with the antioxidant phenotype observed in the present study, but they should be interpreted only as a structural hypothesis rather than evidence that the Keap1/Nrf2 pathway was activated in vivo. Direct evidence would require measurement of Keap1-Nrf2 dissociation, nuclear Nrf2 accumulation, ARE-driven transcription, and downstream proteins such as HO-1, NQO1, and GCLC in liver or intestinal tissues [34,35].

HFD-induced obesity is typically accompanied by dyslipidemia and ectopic lipid accumulation in the liver [36]. SCP intervention reduced epididymal and perirenal fat mass and improved serum lipid profiles; representative liver images also suggested improved hepatic steatosis-related morphology. Importantly, all obese groups were switched from HFD to a normal diet during the 4-week intervention phase. Therefore, the most appropriate interpretation is that SCPs provided additional benefits beyond diet normalization, rather than independently reversing obesity under continued HFD exposure. This distinction is important because final body weight did not differ significantly between SCP-treated groups and the MOD group, whereas adipose tissue mass, lipid markers, and liver histology improved. Thus, the present findings support additional improvements in selected metabolic and hepatic readouts during dietary correction, rather than a broad anti-obesity effect. Because glucose tolerance, insulin tolerance, fasting insulin, HOMA-IR, hepatic triglyceride content, inflammatory cytokines, and energy expenditure were not assessed, the term “metabolic dysfunction” should be interpreted within the limited scope of fat-pad mass, serum biochemical markers, and representative hepatic morphology.

Intestinal barrier impairment is a key contributor to obesity-related metabolic dysfunction [37]. HFD-associated oxidative stress and dysbiosis can damage the epithelial barrier, deplete goblet cells, and increase endotoxin translocation, thereby aggravating hepatic inflammation and steatosis through the gut–liver axis. In the present study, SCPs were associated with improved colonic morphology, increased goblet-cell/mucin-associated staining, reduced serum DAO, and decreased hepatic LPS. These findings support improvements in selected barrier-associated readouts, but they do not establish complete restoration of intestinal barrier integrity. Similarly, reduced hepatic LPS is compatible with decreased gut-derived endotoxin exposure, but it does not by itself prove gut–liver axis regulation. Functional permeability assays such as FITC-dextran flux and analyses of tight-junction proteins such as ZO-1, occludin, and claudins would be required to confirm barrier restoration and strengthen the gut–liver axis hypothesis [38,39].

The gut microbiota is an important mediator of host energy homeostasis and intestinal barrier regulation [40]. SCPs-H administration was associated with partial shifts in HFD-altered microbiota composition, particularly in taxa previously associated with lipid metabolism or SCFA-related microbial profiles. Correlation analysis indicated that these genera were associated with serum lipid profiles, liver function markers, and barrier-related indices. These correlations are biologically plausible but should not be interpreted as causal. Further studies integrating targeted metabolomics or microbiota-transfer experiments would help clarify whether these compositional changes reflect functional microbial effects [41,42].

The in vitro digestion-fermentation and LS174T goblet cell model provided supportive evidence that fermentation products generated in the presence of digested SCPs improved oxidative-stress and MUC2-related readouts under H_2_O_2_ challenge [22,43]. Compared with MOD fermentation products, MOD-SCPs’ fermentation products improved cell viability, selected antioxidant indices, ROS fluorescence, PAS staining, and MUC2 levels. These findings are consistent with the hypothesis that SCP digestion and fermentation may alter the biological activity of fermentation products. However, LS174T cells are colorectal cancer-derived goblet-like cells and do not fully represent normal intestinal epithelium. In addition, the fermentation products were used as unfractionated mixtures after autoclaving and lyophilization, and their chemical composition was not characterized. Therefore, these results should be interpreted as in vitro functional readouts rather than direct evidence that SCP-derived microbial metabolites protect the mucus barrier in vivo. Future metabolomic profiling should identify SCFAs, bile acids, indoles, amino-acid metabolites, and peptide fragments that may contribute to these effects.

Overall, the present study suggests that SCP administration was associated with additional improvements in selected adipose, serum lipid, hepatic antioxidant, colon barrier-associated, and microbiota-related readouts during diet normalization. The in vitro fermentation results further indicate that fermentation products generated in the presence of digested SCPs may improve oxidative-stress and MUC2-related readouts in LS174T goblet-like cells. These findings provide mechanistic clues but do not demonstrate causality or confirm in vivo protection of the intestinal barrier. Further studies are required to directly validate Keap1/Nrf2 pathway activation, identify the active peptide- and fermentation-derived metabolites, and clarify the causal role of specific microbial taxa.

To place the mouse doses in a translational context, the SCP doses used in this study, 400 and 800 mg/kg body weight/day, were converted to approximate human-equivalent doses using body-surface-area normalization [44]. Using Km values of 3 for mice and 37 for adult humans, these doses correspond to approximately 32.4 and 64.9 mg/kg/day, respectively, equivalent to approximately 1.9 and 3.9 g/day for a 60-kg adult. These gram-level intakes may be practically achievable for a standardized food-derived peptide hydrolysate. However, this calculation provides only dose contextualization and should not be interpreted as a recommended human intake level or evidence of comparable exposure to active peptide species in humans. Digestion, absorption, microbiota-dependent fermentation, product standardization, batch reproducibility, allergenicity, and long-term safety remain to be evaluated. Therefore, the doses used in this study should be regarded as exploratory preclinical doses.

The study’s strengths lie in its integrated experimental design, which combined peptide characterization, chemical and cellular antioxidant assays, an HFD-induced obesity model during diet normalization, colon histology, 16S rRNA sequencing, and an in vitro digestion-fermentation/LS174T cell model. Nevertheless, several limitations should be acknowledged. First, because all obese groups were switched from the HFD to a normal diet and final body weight did not differ significantly between SCP-treated mice and the MOD group, the observed improvements should be interpreted as additional benefits during dietary correction rather than independent reversal of obesity. Second, mechanistic evidence remains indirect, as Keap1/Nrf2 pathway activation was not directly validated, intestinal permeability was not assessed using functional assays, and microbiota causality was not established. Third, histological evidence was descriptive. Although slides were coded and representative images were reviewed by investigators blinded to group allocation, formal semi-quantitative histological scoring, adipocyte-size quantification, hepatic triglyceride measurement, Oil Red O-positive area quantification, colon injury scoring, goblet-cell counting, and AB-PAS-positive area quantification were not performed. Fourth, although cumulative food intake was presented and SCP-treated groups did not show lower food intake than the MOD group, food intake alone does not fully capture energy balance because energy expenditure was not assessed. Finally, SCPs are a complex hydrolysate, fermentation products were not chemically characterized, only male mice were used, and long-term safety, allergenicity, dosing feasibility, batch reproducibility, and human relevance remain to be evaluated.

## 5. Conclusions

In conclusion, SCPs prepared from *Solenocera crassicornis* were associated with lower fat-pad mass, improved serum lipid profiles, improved hepatic steatosis-related morphology, enhanced hepatic antioxidant status, and improved selected intestinal barrier-associated readouts in HFD-induced obese mice during diet normalization. In vitro digestion-fermentation data further indicated that fermentation products generated in the presence of digested SCPs improved oxidative stress and MUC2-related readouts in LS174T goblet-like cells. These findings support further investigation of SCPs as standardized marine protein hydrolysates. However, the active peptide and fermentation-product components, direct Keap1/Nrf2 and gut–liver axis mechanisms, long-term efficacy, safety, dosing feasibility, and human relevance remain to be established.

## Data Availability

The data supporting the findings of this study are available from the corresponding author upon reasonable request.

## Supplementary Materials

The following supporting information can be downloaded at https://www.mdpi.com/article/10.3390/nu18122029/s1. Table S1: Composition of the basal fermentation medium; Table S2: Identified peptide sequences of SCPs; Table S3: Free amino acid composition of SCPs; Table S4: Final body weights of mice after the intervention; Table S5: Organ and adipose tissue weights of mice; Figure S1: Cumulative food intake during the 4-week administration period.

## Abbreviations

The following abbreviations are used in this manuscript:

SCPs	*Solenocera crassicornis* peptides
HFD	high-fat diet
ROS	reactive oxygen species
LPS	lipopolysaccharide
TG	triglyceride
TC	total cholesterol
LDL-C	low-density lipoprotein cholesterol
HDL-C	high-density lipoprotein cholesterol
ALT	alanine aminotransferase
AST	aspartate aminotransferase
T-AOC	total antioxidant capacity
SOD	superoxide dismutase
GSH-Px	glutathione peroxidase
CAT	catalase
MDA	malondialdehyde
Keap1	Kelch-like ECH-associated protein 1
Nrf2	Nuclear factor erythroid 2-related factor 2
UPLC-Q-TOF-MS/MS	ultra-performance liquid chromatography coupled with quadrupole time-of-flight tandem mass spectrometry
EDTA	ethylenediaminetetraacetic acid
MTT	3-(4,5-dimethylthiazol-2-yl)-2,5-diphenyltetrazolium bromide
QIIME	Quantitative Insights Into Microbial Ecology
PDB	Protein Data Bank
HO-1	heme oxygenase-1
NQO1	NAD(P)H quinone oxidoreductase 1
AMPK	AMP-activated protein kinase
PPAR	peroxisome proliferator-activated receptor
