# Supplementary material for: Marine Peptides from Solenocera crassicornis Are Associated with Improved Metabolic, Hepatic, and Intestinal Markers During Diet Normalization in HFD-Induced Obese Mice
Source: Nutrients. 2026 Jun 22;18(12):2029. doi: 10.3390/nu18122029 (PMC13305807; doi:10.3390/nu18122029)
Supplement: Supplementary file 1 [file nutrients-18-02029-s001.zip › nutrients-4333082-supplementary.pdf]

Supplementary Materials

Table S1. Composition of the basal fermentation medium

Component	Amount
Tryptone	2 g
Yeast extract	2 g
Tween 80	2 mL
L-Cysteine hydrochloride	0.5 g
Bile salts	0.5 g
Hemin	0.02 g
Vitamin K1	10 μL
Resazurin	0.001 g
Lactulose	10 g
NaCl	0.1 g
K_2HPO_4	0.04 g
$KH_2 PO_4$	0.04 g
$MgSO_4 \cdot 7H_2O$	0.01 g
$CaCl_2$	0.01 g
$NaHCO_3$	2 g
Distilled water	1 L

Table S2. Main peptides in SCPs

**Peptide**	**ALC (%)**	**Length**	***m/z***	***z***	**RT**	**Area**	**Mass**
ELDGGAP	99	7	658.3061	1	4.59	1.36E+04	657.2969
APPPPP	98	6	575.3188	1	4.5	6.49E+03	574.3115
TAMPN	98	5	549.2309	1	4.81	1.23E+04	548.2264
HPPPP	98	5	544.2667	1	4.57	1.71E+03	543.2805
EVLPP	98	5	554.3201	1	5.47	1.11E+03	553.3112
GADPL	97	5	472.2424	1	4.56	6.77E+03	471.2329
DGFPN	97	5	549.2311	1	4.81	1.15E+04	548.2231
MLPLP	97	5	586.3088	1	5.19	1.69E+04	585.3196
MPLPP	97	5	554.3201	1	5.47	1.04E+03	553.2934
AAPLE	96	5	500.2735	1	5.03	8.50E+02	499.2642
LALL	96	4	429.3079	1	6.1	7.67E+03	428.2999
AGAAGTPE	95	8	673.3183	1	3.98	5.35E+02	672.3079
CGAAKAPQ	95	8	381.1877	2	4.63	6.20E+02	760.3537
KAAP	95	4	386.2523	1	0.7	6.09E+02	385.2325
LPLP	95	4	439.2917	1	5.71	1.10E+03	438.2842
ELPP	95	4	228.1362	2	5.29	1.64E+03	454.2427
MVEVM	95	5	312.6328	2	5.19	1.33E+02	623.2659

Table S3. Amino acid composition of SCPs

Amino acid	Content (mg/g)
Aspartate (Asp)	12.126
Threonine (Thr)	6.407
Serine (Ser)	10.824
Glutamic acid (Glu)	16.271
Glycine (Gly)	24.451
Alanine (Ala)	9.663
Cystine (Cys)	7.049
Valine (Val)	9.293
Methionine (Met)	10.003
Isoleucine (Ile)	7.789
Leucine (Leu)	20.59
Tyrosine (Tyr)	23.467
Phenylalanine (Phe)	25.455
Histidine (His)	5.688
Lysine (Lys)	17.601
Arginine (Arg)	34.627
Proline (Pro)	5.517
TAA	246.821
∑EAA	97.138
∑NEAA	149.683

Note: EAA, essential amino acids; NEAA, non-essential amino acids; TAA, total amino acids.

Table S4. Body weight of mice after 4 weeks of intervention

Group	n	Body weight after modeling (g)	Final weight (g)
ND	10	26.604±0.99[b]	27.150±1.25[b]
MOD	10	32.181±2.08[a]	29.892±1.11[b]
PC	10	32.051±2.06[a]	28.353±2.00[b]
SCPs-L	10	31.011±2.26[a]	27.438±1.35[b]
SCPs-H	10	32.126±3.03[a]	27.204±1.82[b]

Values are expressed as mean ± SD. Different superscript letters within the same column indicate significant differences ($p < 0.05$).

Table S5. Weight of mice organs and adipose tissue

Group	Liver (g)	Kidney (g)	Spleen (g)	Epididymal fat (g)	Perirenal fat (g)
ND	1.395±0.09[a]	0.321±0.02[a]	0.070±0.02[a]	0.651±0.12[b]	0.160±0.06[b]
MOD	1.388±0.09[a]	0.335±0.02[a]	0.069±0.01[a]	1.119±0.34[a]	0.309±0.10[a]
PC	1.384±0.16[a]	0.329±0.03[a]	0.071±0.07[a]	0.774±0.13[b]	0.199±0.03[b]
SCPs-L	1.277±0.08[b]	0.330±0.04[a]	0.075±0.01[a]	0.776±0.17[b]	0.211±0.09[b]
SCPs-H	1.248±0.09[b]	0.321±0.03[a]	0.074±0.01[a]	0.775±0.19[b]	0.195±0.07[b]

Values are expressed as mean ± SD. Different superscript letters within the same column indicate significant differences ($p < 0.05$).

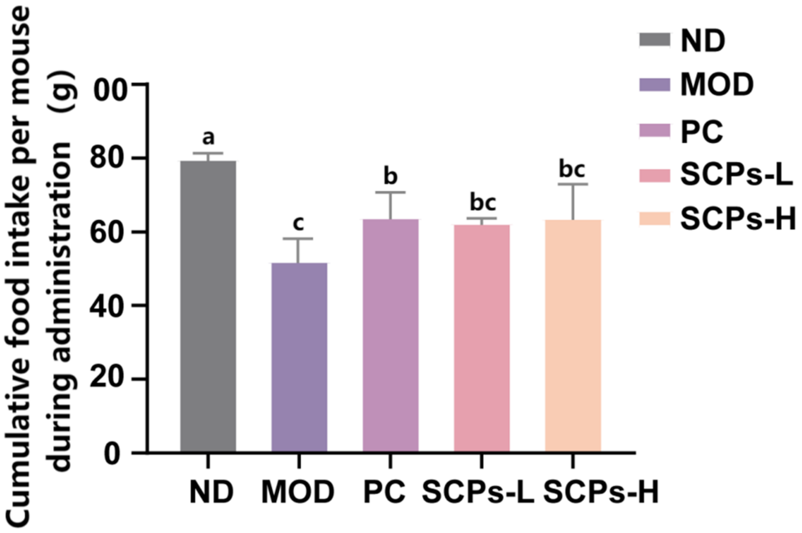

Figure S1. Cumulative food intake during the 4-week administration period. Data are expressed as mean ± SD. Different letters indicate significant differences among groups ($p < 0.05$).
